# Exosomal miR-1260b derived from non-small cell lung cancer promotes tumor metastasis through the inhibition of HIPK2

**DOI:** 10.1038/s41419-021-04024-9

**Published:** 2021-07-28

**Authors:** Dong Ha Kim, Hyojeong Park, Yun Jung Choi, Myoung-Hee Kang, Tae-Keun Kim, Chan-Gi Pack, Chang-Min Choi, Jae Cheol Lee, Jin Kyung Rho

**Affiliations:** 1 Asan Institute for Life Sciences, Asan Medical Center, University of Ulsan, College of Medicine, Seoul, 05505 South Korea; 2 Department of Biomedical Sciences, Asan Medical Center, AMIST, University of Ulsan, College of Medicine, Seoul, 05505 South Korea; 3 Department of Convergence Medicine, Asan Medical Center, University of Ulsan, College of Medicine, Seoul, 05505 South Korea; 4 Department of Pulmonology and Critical Care Medicine, Asan Medical Center, University of Ulsan, College of Medicine, Seoul, 05505 South Korea; 5 Department of Oncology, Asan Medical Center, University of Ulsan, College of Medicine, Seoul, 05505 South Korea

**Keywords:** Non-small-cell lung cancer, Metastasis, miRNAs

## Abstract

Tumor-derived exosomes (TEXs) contain enriched miRNAs, and exosomal miRNAs can affect tumor growth, including cell proliferation, metastasis, and drug resistance through cell-to-cell communication. We investigated the role of exosomal miR-1260b derived from non-small cell lung cancer (NSCLC) in tumor progression. Exosomal miR-1260b induced angiogenesis by targeting homeodomain-interacting protein kinase-2 (HIPK2) in human umbilical vein endothelial cells (HUVECs). Furthermore, exosomal miR-1260b or suppression of HIPK2 led to enhanced cellular mobility and cisplatin resistance in NSCLC cells. In patients with NSCLC, the level of HIPK2 was significantly lower in tumor tissues than in normal lung tissues, while that of miR-1260b was higher in tumor tissues. HIPK2 and miR-1260b expression showed an inverse correlation, and this correlation was strong in distant metastasis. Finally, the expression level of exosomal miR-1260b in plasma was higher in patients with NSCLC than in healthy individuals, and higher levels of exosomal miR-1260b were associated with high-grade disease, metastasis, and poor survival. In conclusion, exosomal miR-1260b can promote angiogenesis in HUVECs and metastasis of NSCLC by regulating HIPK2 and may serve as a prognostic marker for lung cancers.

## Introduction

Extracellular vesicles (EVs) are lipid bilayer-enclosed particles that are secreted by almost all cell types of mammalian organisms. These vesicles are broadly classified into apoptotic bodies, microvesicles, and exosomes according to their size and cellular origin. EVs contain intracellular DNA, mRNA, miRNA, and proteins, which can be transported to other cells [1–3]. Although EVs were initially regarded as “garbage bags” in eliminating unwanted substances from cells, many studies have revealed their function as tools for cell-to-cell communication. To date, several studies have identified the role of tumor-derived exosomes or EVs in various stages of tumor progression, including development [4], angiogenesis [5, 6], evasion of immune surveillance [7–9], metastasis [10, 11], and acquisition of aggressive phenotypes and multidrug resistance [12, 13].

miRNAs represent an extensive class of small, noncoding RNAs that play important roles in regulating mRNA by degrading it and adjusting protein levels. miRNAs are enriched in exsosomes, as confirmed in our previous study [14–17]. Many studies have shown that exosomal miRNAs can get transferred to neighboring and distant cells [18–20] and play functional roles in tumor growth. Recently, some reports have demonstrated that miR-1260b is associated with chemosensitivity [21], lymph node metastasis [22], cell proliferation and apoptosis [23], and cellular mobility in various tumor cells [24]. Xia et al. reported that the transfer of exosomal miR-1260b can promote cell invasion in lung adenocarcinoma [25] and suggested the role of miR-1260b as a diagnostic or prognostic marker in various tumor types. However, further validation is required for the application of miR-1260b as a clinical biomarker.

Homeodomain-interacting protein kinase-2 (HIPK2) is a serine/threonine kinase belonging to the dual-specificity tyrosine phosphorylation-regulated kinase family of protein kinases [26]. HIPK2 is considered a tumor suppressor that modulates growth and apoptotic cellular responses. HIPK2 can also promote apoptosis by targeting multiple proteins, including p53, p73, antiapoptotic trans-repressor C-terminal binding protein, mouse double minute 2, and scaffold Axin [27–32]. In addition, HIPK2 plays an important roles in the inhibition of angiogenesis by regulating vascular endothelial growth factor (VEGF), Siah-1, Siah-2, WD repeat and SOCS box-containing protein 1, and hypoxia-inducible factor 1 in hypoxic environment [33–38]. Thus, HIPK2 is a promising target for anticancer therapies.

Our previous study identified that some miRNAs, including miR-619-5p, were associated with angiogenesis and metastasis and some, including miR-1260b, were enriched in non-small cell lung cancer (NSCLC)-derived exosomes [17]. Thus, this study was designed to investigate the function of exosomal miR-1260b by investigating how exosomal miR-1260b induced angiogenesis in endothelial cells and cellular mobility in NSCLC cells by targeting HIPK2 to determine whether exosomal miR-1260b could serve as a predictive indicator for metastasis in NSCLC.

## Materials and methods

### Cell culture

The human NSCLC cell lines A549 and Calu-1 were purchased from American Type Culture Collection (ATCC; Rockville, MD), and PC-9 cell line was provided by Dr Kazuto Nishio (National Cancer Center Hospital, Tokyo, Japan). NSCLC cell lines were maintained in RPMI1640 with 100 U/mL penicillin, 100 mg/mL streptomycin, and 10% fetal bovine serum (FBS). Human umbilical vein endothelial cells (HUVECs) were purchased from ATCC and cultured between passages 1 and 5 in Medium 200 (Gibco, ON, Canada) supplemented with low serum growth supplement, 5% FBS, 100 U/mL penicillin, and 100 mg/mL streptomycin (Gibco). All cell lines were cultured in a 95% humidified incubator at 37 °C with 5% CO_2_ and tested to be mycoplasma-free using the MycoProbe Mycoplasma Detection Kit (R&D Systems, Minneapolis, MN) before freezing.

### Tube formation assay

An endothelial tube formation assay was performed as described previously [17]. Briefly, HUVECs (0.5 × 10^5^ cells/well) were seeded into 12-well plates, which were precoated with Matrigel (Corning Life Science, Corning, NY), and transfected with miRNA or treated with exosomes at 37 °C with 95% humidified air and 5% CO_2_. After 3–6 h, the cells were stained with Calcein AM dye and visualized on the Invitrogen EVOS M5000 Imaging System (Thermo Fisher Scientific, CA). The total tube lengths were calculated using Angiogenesis Analyzer for Image J software.

### Generation of stable cell lines

To generate a lentiviral vector expressing miR-1260b (MI0014197), the miR-1260b (Genecopoeia, HmiR0803-MR03, Rockville, MD) and miR-1260b inhibitor (Genecopoeia, HmiR-AN1496-AM03) sequences were cloned into a lentiviral vector. The HIPK2 expression vector (Origene, RC220278, Rockville, MD) and HIPK2 shRNA constructs (Origene, TR304106) were cloned into a retroviral vector. Cells were incubated with culture medium-diluted virus supernatant in the presence of 8 μg/mL polybrene (Sigma-Aldrich, St. Louis, MO). For stable infection experiments, G418 (Sigma-Aldrich) or Puromycin (Thermo Fisher, Waltham, MA) was added for stable clone selection.

### Exosome isolation

The A549 cell line and its stable cell lines (miR-1260b-O/E and anti-miR-1260b-O/E) were washed with PBS and grown in serum-free RPMI1640. For exosome isolation, the conditioned medium was collected from cells cultured in dishes for 48 h. In the first step, cellular debris were removed from the conditioned medium at 300 × *g* for 10 min, 2000 × *g* for 10 min, and 10,000 × *g* for 30 min at 4 °C. The supernatants were collected without disturbing at 100,000 × *g* for 70 min at 4 °C. The pellets were washed with PBS, ultracentrifuged, and resuspended in PBS. Thawed plasma samples were isolated using the same method. Exosome isolations were performed as described previously [39].

### Negative staining electron microscopy

Negative staining analysis of exosome was performed as described previously [39]. Briefly, purified exosomes were fixed in 2% paraformaldehyde. Nickel transmission electron microscopy (TEM) grids, 200 mesh with a formvar/carbon film, were floated on a drop of the fractions of exosomes. The grids were stained with 2% uranyl acetate and imaged using TEM (Hitachi H7600, Japan) at 80 kV.

### Nanoparticle tracking analysis

Nanoparticle tracking analysis (NanoSight NS300, Malvern Instruments Ltd, Malvern, UK) was used to measure the number and size distribution of exosomes. Purified exosomes were diluted 100- to 500-fold in PBS, and readings were imaged thrice for 60 s at room temperature. Data were analyzed using the nanoparticle tracking analysis software (NTA version 2.3 build 0017).

### 3′-UTR luciferase reporter constructs and luciferase assays

**Fig. 1:**
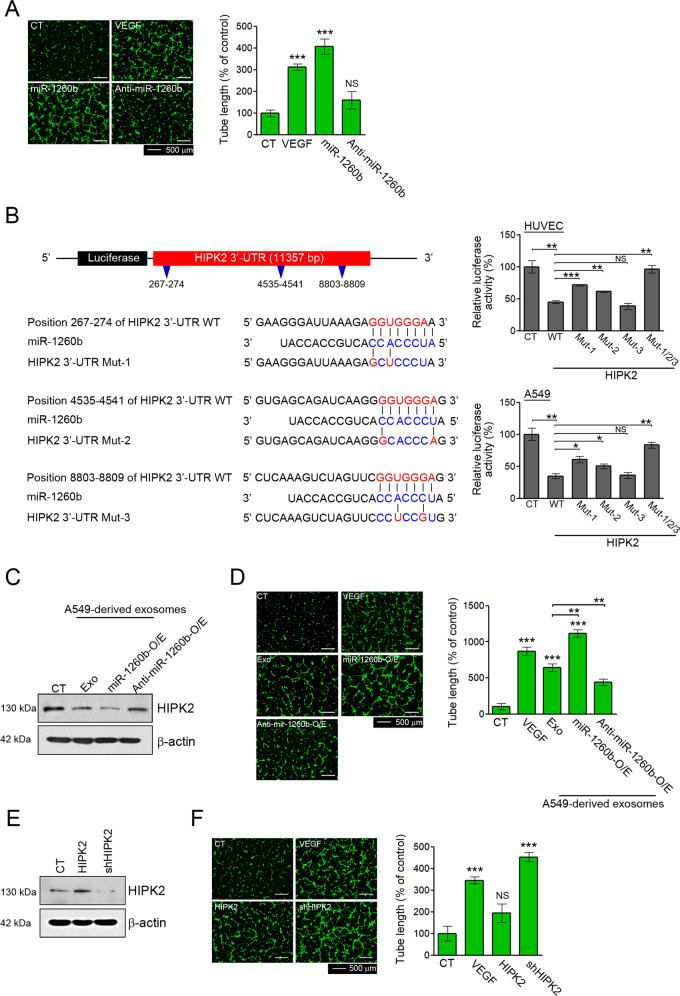
Effects of exosomal miR-1260b on angiogenesis via direct modulation of HIPK2. **A** HUVECs were treated with 40 ng/mL VEGF, a 50 nM miR-1260b mimic, and a miR-1260b inhibitor. Effect of miR-1260b on the tube formation ability was determined by tube length. Tube lengths were measured using ImageJ software. **B** Schematic diagram of the putative miR-1260b-binding site within the 3′UTR of HIPK2. The seed sequence of miR-1260b matches three predicted target sites (nucleotides 267–274, 4535–4541, and 8803–8809; red). Five nucleotides within each target site complementary to the seed sequence (nucleotides 2–7 of miRNA) of miR-1260b were mutated in the HIPK2 3′UTR-mutant plasmids including single (Mut-1: 267–274, Mut-2: 4535–4541, and Mut-3: 8803–8809) or triple (Mut-1/2/3: 267–274, 4535–4541, and 8803–8809) mutants. The number indicates the position of the nucleotides in the wild-type (WT) sequence of the HIPK2 3′UTR site. For the dual-luciferase assay, luciferase activities of plasmids with WT or Mut sequence of HIPK2 were assessed in HUVECs and A549 cells co-transfected with miR-1260b mimic, and then Renilla luciferase activity was calculated as the luciferase activity ratio of firefly to Renilla luciferase. **C**–**F** HUVECs were treated with 40 ng/mL VEGF, 50 μg of exosomes derived from A549, and their stable cell lines (miR-1260b-O/E and Anti-miR-1260b), lentiviral HIPK2 or shHIPK2. **D**, **F** Tube lengths were measured using ImageJ software. **C**, **E** HIPK2 expression was confirmed by western blotting. All data are reported as the mean ± standard deviation. *P < 0.05, **P < 0.005, ***P < 0.0005 compared with the control group.

The 3′-UTR sequence or the mutant sequence of HIPK2 (NM_022740) was cloned into the predicted miR-1260b binding sites using the pEZX-MT06 Renilla/firefly dual-luciferase reporter plasmid (GeneCopoeia, HmiT067235, Rockville, MD). Mutant constructs were generated by single (Mut-1:267–274, Mut-2:4535–4541, and Mut-3:8803–8809) or triple (Mut-1/2/3: 267–274, 4535–4541, and 8803–8809) site-specific mutations in the seed target sites (Fig. 1). The Dual-Luciferase Reporter Assay System (Promega, Madison, WI) was used to perform luciferase assays. Firefly luciferase activity was normalized by Renilla luciferase activity, and both activities were measured using the 2030 multilabel reader VICTOR™ X3 (Perkin Elmer, Waltham, MA, USA).

### Migration and invasion assays

Transwell migration and invasion assays (Corning Life Science) were performed using 24-well plates with polycarbonate membrane inserts with an 8 μm pore size. Briefly, the cells supplemented with serum-free medium were seeded into the Matrigel-coated chambers (for invasion) or Type I collagen-coated chambers (for migration). The lower chambers contained RPMI1640 with 10% FBS. After incubation for 24 h, the migrated or invaded cells were fixed and stained using the Hemacolor^®^ rapid staining kit (Merck, Darmstadt, Germany). The cells at the bottom of the membrane were counted under a light microscope.

### Tail vein metastasis model

To verify that miR-1260b affects lung metastasis, A549 cells were infected with lentiviruses carrying the luciferase reporter gene and then transfected with miR-1260b. A total of 1 × 10^6^ cells were resuspended in 100 μl PBS and injected into the tail veins of severe combined immunodeficiency mice (6 weeks of age) using 31-gauge insulin syringes. Mice were injected with 150 mg/kg D-luciferin potassium salt (Caliper Life Sciences, Hopkinton, MA) and monitored weekly using the IVIS system (Xenogen, Alameda, CA). Metastasis in vivo was measured via bioluminescence imaging.

### Western blotting

The total proteins from cells or exosomes were extracted using EBC lysis buffer and quantified using the Bradford method. Approximately 20 mg of protein was separated by SDS-PAGE and transferred to PVDF membranes (Invitrogen) for western blotting. Membranes were probed using antibodies against HIPK2 (#5091, 1:1000, Cell Signaling Technologies, Danvers, MA), HSP70 (BD610607, 1:4000, BD Biosciences, San Diego, CA), CD9 (ab92726, 1:1000, Abcam, Cambridge, UK), TSG101 (ab125011, 1:1000, Abcam), calnexin (ab22595, 1:1000, Abcam), and β-actin (SC47778,1:2000, Santa Cruz Biotechnology, Santa Cruz, CA) as the primary antibodies.

### Cell apoptosis assay

The FITC Annexin V Apoptosis Detection Kit (BD Biosciences) was used to quantitatively determine cell apoptosis according to the manufacturer’s instructions. Cells were transfected with miR-1260b or treated with exosomes and grown in 5% FBS RPMI medium containing a final concentration of 5 or 10 μM cisplatin (Sigma-Aldrich, St. Louis, MO, USA) for 48 h. Cells were stained with FITC-conjugated Annexin V and PI for 20 min at 37 °C in the dark. Flow cytometry of samples was performed using the BD Canto II cytometer (BD Biosciences). Experiments were performed in triplicate.

### Clinical specimens

Human NSCLC cells and their matched adjacent noncancerous lung tissues were collected from 124 paired patients at Asan Medical Center between 2009 and 2016. Clinicopathological characteristics, including TNM stage, are listed in Table S1. Forty-eight patients with and 48 healthy volunteers plasma samples were acquired through collection of whole blood in ethylenediaminetetraacetic acid after obtaining prior consent from each individual. These analyses were approved by the Institutional Review Board of Asan Medical Center (2016-0752, 2018-0462).

### Quantitative real-time reverse transcription polymerase chain reaction (qRT-PCR)

Total RNA was isolated with the RNeasy Miniprep kit (Qiagen) according to the manufacturer’s instructions. Extracted RNA was reverse-transcribed to cDNA using polyadenylated with a poly(A) tailing kit (Ambion, Austin, TX) and poly(T) adaptor before reverse transcription.

The ABI 7900 Real-Time PCR System enabled SYBR-green-based detection. The primers used are listed in Table S2.

### Statistical analysis

Data are presented as mean ± standard deviation. *p* values were determined using unpaired *t*-tests between groups using GraphPad Prism software.

## Results

### Exosomal miR-1260b promotes angiogenesis in HUVECs by directly targeting HIPK2

To confirm the effect of miR-1260b on angiogenesis in HUVECs, we performed transfection with human miR-1260b or its complementary antagonist anti-miR-1260b. We confirmed that these oligonucleotides were taken up by HUVECs (Fig. S1A) and evaluated their effects on tube formation. miR-1260b treatment increased tube formation, whereas anti-miR-1260b treatment showed no effect (Fig. 1A). These results supported the angiogenic effect of miR-1260b in HUVECs.

We predicted 40 potential target genes of miR-1260b using five algorithms (Fig. S2). Among the potential target genes, HIPK2 was selected for further validation because of its well-known role in tumor angiogenesis. HIPK2 was found to have three binding sites for miR-1260b in its 3′ UTR (Fig. 1B). To determine whether HIPK2 was a direct target of miR-1260b, we designed luciferase expression plasmids that included sequences of the wild-type (WT) and mutants (MUT1-3) in three predicted binding sites of miR-1260b on the 3′ UTR of HIPK2. Dual-luciferase reporter analysis showed that the relative luciferase activity of HUVECs and A549 cells was decreased significantly with WT treatments, but these reductions recovered with MUT1 and MUT2 treatments. Furthermore, triple mutation (MUT1/2/3) completely abrogated the effect of miR-1260b (Fig. 1B). These results indicate that miR-1260b directly targets HIPK2.

To further validate the angiogenic effects of exosomal miR-1260b in HUVECs, we isolated exosomes from A549 cells and their stable cell lines (miR-1260b- and anti-miR-1260b-overexpressing A549 cells). Exosomes derived from each cell line were classically confirmed by their size, morphology, and protein markers (Fig. S3). When HUVECs were treated with each exosome, induction of miR-1260b and reduction of HIPK2 were confirmed (Figs. S1B and 1C). In these conditions, treatment with exosomes derived from A549 cells showed increased tube formation; exosomes derived from miR-1260b-overexpressing A549 cells (miR-1260b-O/E) stimulated more tube formation than those derived from A549 cells. Furthermore, exosomes derived from anti-miR-1260b-overexpressing A549 cells or anti-miR-1260b treatment attenuated tube formation by A549 cell-derived exosomes (Figs. 1D and S4). To further validate the role of HIPK2 in angiogenesis, HUVECs were treated with shRNA HIPK2 or an expression vector for HIPK2. Under conditions of HIPK2 suppression or overexpression (Fig. 1E), HIPK2 suppression significantly enhanced tube formation, whereas HIPK2 overexpression showed no effect (Fig. 1F). Thus, exosomal miR-1260b led to enhanced tube formation capacity by reducing HIPK2 protein levels in HUVECs.

### Exosomal miR-1260b promotes migration and invasion of NSCLC cells

**Fig. 2:**
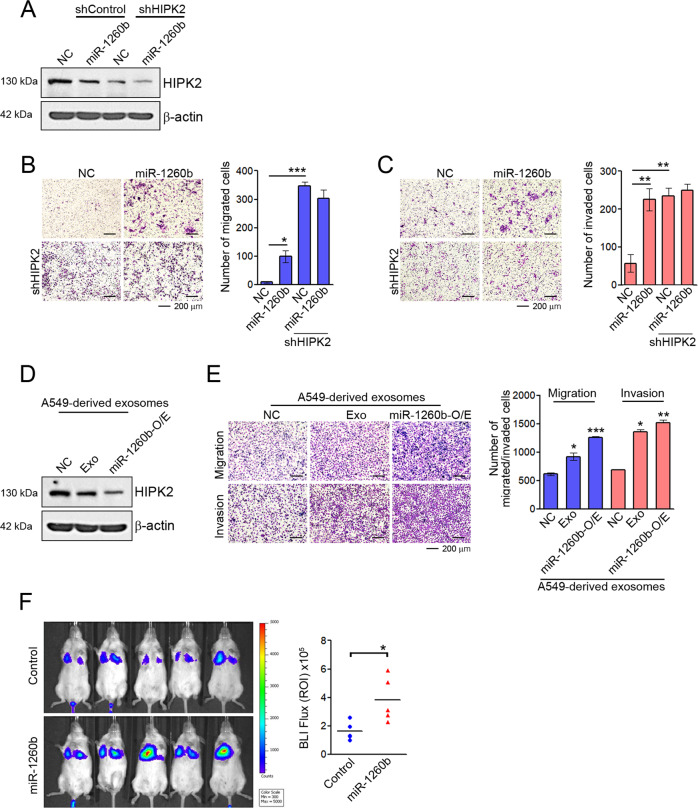
Effects of exosomal miR-1260b on migration and invasion of NSCLC. **A**–**C** A549 cells were transfected with 50 nM control miRNA (NC) or miR-1260b mimic for 48 h following infection of lentiviral shcontrol or shHIPK2. **A** HIPK2 expression was confirmed by western blotting. **B**, **C** Transwell assays were performed to detect changes in migration and invasion abilities. The number of migratory or invading cells was counted for each image field. **D**, **E** PC-9 cells were treated with 50 μg of exosomes derived from A549 and their stable cell lines (miR-1260b-O/E or anti-miR-1260b-O/E). HIPK2 expression was confirmed by western blotting, and the ability of migration and invasion was determined using Transwell assays. Data are reported as the mean ± standard deviation of three independent experiments with five fields counted per experiment. **F** IVIS luciferase in vivo images of lung metastasis. Lung metastasis models by using A549 cells were established as described in “Materials and methods.” Luciferase activities were determined by bioluminescent imaging (BLI) at 2 weeks after injection of the indicated cells. *P < 0.05, **P < 0.005, ***P < 0.0005 compared with the control group.

Some studies have suggested that miR-1260b plays a role as a regulator of tumor metastasis [24, 40, 41]. To further investigate the biological consequences of miR-1260b/HIPK2-mediated changes in NSCLC, we searched the ability to cellular mobility by miR-1260b according to HIPK2 expression. Suppression of HIPK2 expression by shRNA or miR-1260b was confirmed by western blotting (Fig. 2A). Although miR-1260b treatment did not affect cell proliferation in NSCLC cells (Fig. S5), HIPK2 suppression by shRNA or miR-1260b significantly enhanced the migration and invasiveness of NSCLC cells, whereas miR-1260b did not affect the ability to cellular mobility under reduced HIPK2 expression (Figs S6 and 2B, C). These results were similar to those obtained by HIPK2 suppression by treatment with A549-derived exosomes or exosomes derived from miR-1260b-overexpressing A549 cells (Fig. 2D, E). Furthermore, the introduction of miR-1260b resulted in higher lung metastatic capacity than A549 cells following tail vein injection (Fig. 2F). Collectively, our data demonstrate that exosomal miR-1260b promotes the cellular mobility of NSCLC cell lines by regulating HIPK2 expression.

### Exosomal miR-1260b impairs the sensitivity of NSCLC cells to cisplatin

**Fig. 3:**
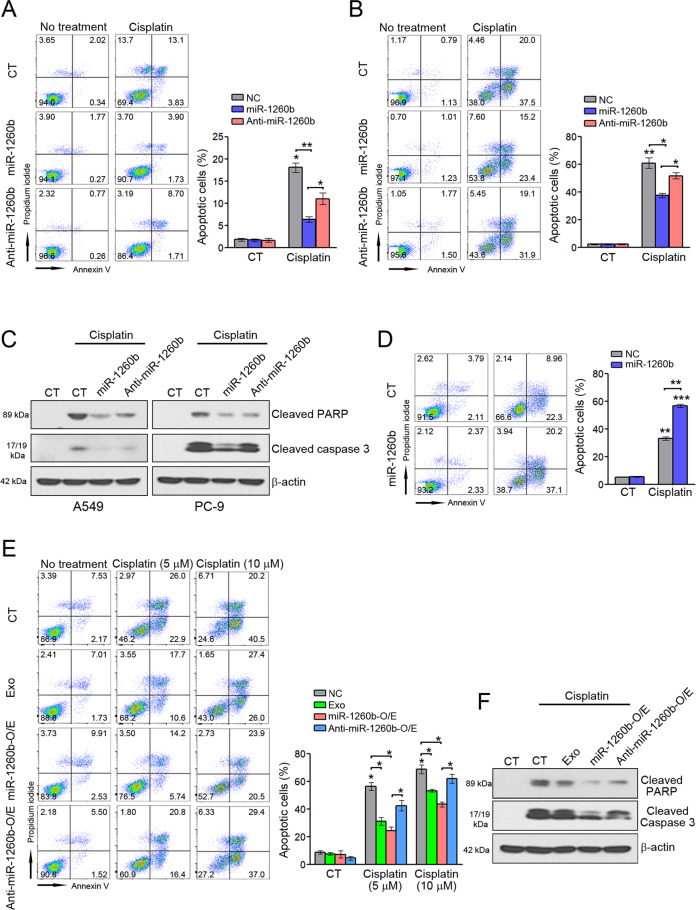
Effect of exosomal miR-1260b on cisplatin-induced apoptosis. **A** A549, **B** PC-9, and **D** Calu-1 cells were transfected with 50 nM control miRNA (CT), miR-1260b mimic, or anti-miR-1260b mimic for 24 h and then treated with 10 μM cisplatin for 48 h. Apoptosis was measured by flow cytometry. **C** Cleaved PARP and caspase-3 were detected by western blotting. **E** PC-9 cells were treated with 50 μg exosomes from A549, miR-1260b-overexpressing A549, or anti-miR-1260b-overexpressing A549 cells and treated with the indicated doses of cisplatin for 48 h. Apoptosis was measured by flow cytometry. **F** Cleaved PARP and caspase-3 were detected by western blotting. The results are reported as the mean ± standard deviation of three independent experiments. *P < 0.05, **P < 0.005, ***P < 0.0005 compared with the control group.

Tumor-derived exosomes can transfer multidrug resistance-associated protein, mRNA, and miRNA to recipient cells [42–44], which leads to resistance to anticancer drugs. We investigated whether miR-1260b affects the sensitivity of NSCLC cells to cisplatin. Flow cytometry revealed that miR-1260b treatment inhibited cisplatin-induced apoptosis in A549 and PC-9 cells, whereas anti-miR-1260b treatment attenuated the inhibition of cisplatin-induced apoptosis by miR-1260b (Fig. 3A, B). Consistent with these results, apoptosis signaling, including PARP and caspase-3, was confirmed by western blotting (Fig. 3C). Unlike the results of A549 and PC-9 cells, the opposite pattern was found in Calu-1 cells (Fig. 3D). To validate whether exosomal miR-1260b leads to resistance to cisplatin, as shown in the aforementioned results, we treated the cells with A549-derived exosomes, exosomes derived from miR-1260b-overexpressing, or anti-miR-1260b-overexpressing A549 cells and found that treatment of exosomes containing miR-1260b inhibited cisplatin-induced apoptosis. These effects were more significant in cells treated with exosomes derived from miR-1260b-overexpressing A549 cells than in A549-derived exosomes. In addition, exosomes derived from anti-miR-1260b-overexpressing A549 cells attenuated the inhibition of cisplatin-induced apoptosis by miR-1260b (Fig. 3E), which were also confirmed by apoptosis signaling (Fig. 3F). These findings suggest that exosomal miR-1260b reduces the sensitivity of NSCLC cells to cisplatin.

### Clinical implications of HIPK2 and miR-1260b in patients with NSCLC

**Fig. 4:**
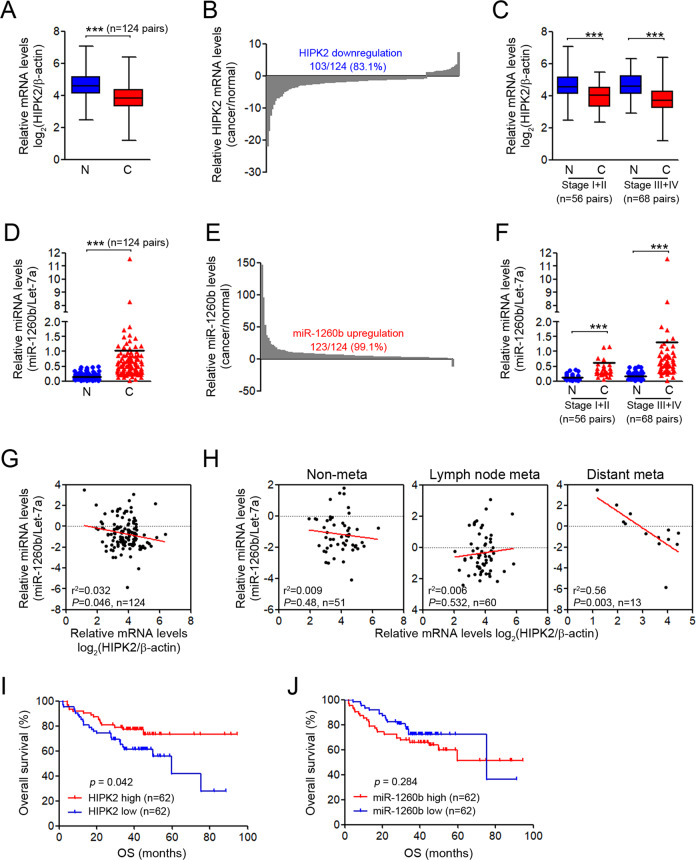
Relationship between HIPK2 and miR-1260b expression in NSCLC tissues. Expression levels of miR-1260b and HIPK2 were determined by qRT-PCR assay in 124 pairs of NSCLC and adjacent normal tissue samples. **A** Box plot expression of HIPK2 mRNA levels in paired NSCLC samples. **B** Fold change in HIPK2 mRNA in 124 cancer tissues divided by that in paired adjacent normal tissues. **C** Comparisons of HIPK2 mRNA expression at different pathological stages. **D** Dot plot of miR-1260b expression in paired NSCLC samples. **E** Fold change in miR-1260b in 124 cancer tissues divided by that in paired normal tissues. **F** Comparisons of miR-1260b expression at different pathological stages. **G** Scatter plot showing the correlation between HIPK2 mRNA and miR-1260b expression in tumor samples and **H** each TNM subset. **I**, **J** Kaplan–Meier survival curve according to the categories of low and high expression of HIPK2 mRNA and miR-1260b. ***P < 0.0005.

To verify the relationship between miR-1260b and HIPK2 and their clinical meaning, we analyzed the expression levels of miR-1260b and HIPK2 in 124 paired NSCLC tissues and adjacent noncancerous lung tissues using qRT-PCR. HIPK2 transcripts were significantly decreased in NSCLC tissues compared with corresponding noncancerous lung tissues (83.1%, Fig. 4A, B), whereas miR-1260b expression was much higher in NSCLC tissues than in noncancerous lung tissues (99.1%, Fig. 4D, E). When HIPK2 and miR-1260b were assessed according to the TNM stage (early stages I + II vs. late stages III + IV), HIPK2 was decreased and miR-1260b was enhanced, regardless of the tumor stage (Fig. 4C, F). Our clinical association data revealed that HIPK2 downregulation was significantly associated with distant metastasis (*p* = 0.04) and miR-1260b upregulation was significantly associated with lymph node (*p* = 0.001) and distant (*p* = 0.026) metastasis (Table S1). Consistent with these results, scatter plots revealed a strong inverse correlation between miR-1260b level and HIPK2 expression (Fig. 4G), which was more evident in patients with distant metastasis (Fig. 4H). In additiona, Kaplan–Meier survival analysis revealed that patients with low HIPK2 expression had worse overall survival rates than those with high expression (Fig. 4I). However, no significant difference was observed in survival rates among patients with different expression levels of miR-1260b (Fig. 4J). These results suggest that HIPK2 expression is inversely associated with miR-1260b expression and that HIPK2 is an important prognostic indicator or predictor of metastasis in NSCLC.

### Exosomal miR-1260b is induced in patients with NSCLC

**Fig. 5:**
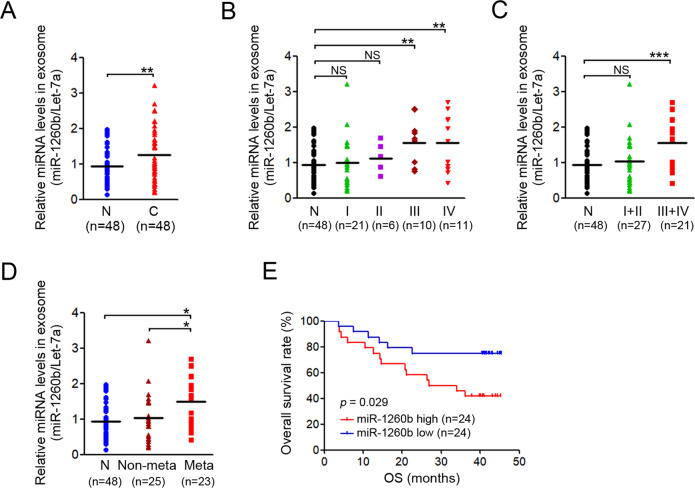
Pathological features of exosomal miR-1260b in NSCLC. **A** Exosomal miR-1260b expression in the plasma of healthy donors (*n* = 48) and patients with NSCLC (*n* = 48). **B** Expression levels of exosomal miR-1260b were analyzed according to different stages, including **C** early (I–II) and late (III–IV) stage or **D** metastatic and nonmetastatic stages of NSCLC. **E** Kaplan–Meier survival curve stratified by high and low exosomal miR-1260b expression levels. *P < 0.05, **P < 0.005, ***P < 0.0005.

We evaluated the level of exosomal miR-1260b in the plasma of healthy donors and patients with NSCLC. In our previous report, we confirmed that let-7a-5p has an equivalent concentration within the exosomes of both healthy donors and patients with NSCLC compared with other miRNAs [17]. Based on these results, we used let-7a-5p as a control to normalize exosomal miR-1260b expression. Expression levels of exosomal miR-1260b were higher in patients with NSCLC than in healthy donors (Fig. 5A) as well as in later TNM stages than in earlier TNM stages (Fig. 5B, C). When comparing patients with and without metastasis, the exosomal miR-1260b expression was significantly increased in those with metastasis (Fig. 5D). In addition, Kaplan–Meier survival analysis showed that patients with high exosomal miR-1260b levels had worse overall survival rates than those with low exosomal miR-1260b levels (Fig. 5E). Taken together, our data suggest that exosomal miR-1260b is highly expressed in patients with metastasis and exosomal miR-1260b is a more powerful biological indicator than cellular miR-1260b as a prognostic indicator or predictor of metastasis in NSCLC.

## Discussion

EVs including exosomes are important players in intercellular communication. Although the definition of an exosome remains unclear, emerging evidence suggests that exosomes are 30–150 nm EVs of endosomal origin, comprising a subset of bioactive molecules, such as DNA, proteins, noncoding RNA (ncRNA), and lipids [45–47]. In cancer biology, these exosomes have recently received attention because tumor exosomes promote disease progression both by contributing to pre-metastatic niche formation and by promoting the evasion of immune surveillance, stimulating angiogenesis, and extracellular matrix degradation [48]. Thus, the modulation of tumor-derived and tumor-associated exosomes may support new therapeutic strategies.

Although exosomes contain various molecules from their original cells, several studies have suggested that exosomal miRNAs promote tumor progression in various model systems [49, 50]. MiRNAs are enriched in exosomes, and we and other authors have demonstrated that certain miRNAs are selectively enriched in cancer-derived exosomes, compared to exosomes derived from normal cells [17, 51–53]. Therefore, these exosomal miRNAs may induce functional changes in various recipient cells, suggesting a significant role of exosomes in the malignant process of tumor development. In the context of tumor progression by tumor-derived exosomes, our data showed that exosomal miR-1260b play various roles in angiogenesis, cellular mobility, and drug resistance. These multiple functions of miR-1260b are possible because tumor-derived exosomes can be transferred to a recipient cell located in the tumor microenvironment. These findings indicate that the modulation of tumor-derived exosomes may be an important factor for treating tumors due to their multiple roles.

One miRNA can modulate the expression of various target mRNAs in different pathways. To date, some studies have demonstrated that miR-1260b is associated with chemosensitivity and metastasis [21, 22]. Xia et al. showed that exosomal miR-1260b promotes cell invasion through the Wnt/β-catenin signaling pathway in lung adenocarcinoma [25]. Consistent with the findings of previous studies, we found that the introduction of miR-1260b or exosomal miR-1260b induced migration and invasion or resistance to cisplatin in NSCLC cells. In addition, although some studies demonstrated that miR-1260b can induce angiogenesis via VEGF secretion in tumor cells [23, 54], our study is the first to show that miR-1260b enhances angiogenesis by targeting HIPK2 in endothelial cells. Thus, we speculate that the effects of miR-1260b on tumor-associated angiogenesis are more potent in in vivo systems. Additional studies are needed to validate these possibilities.

Our data showed that miR-1260b can target HIPK2. HIPK2 is a central regulator of life-and-death decisions and potential tumor suppressor in tumor biology. Inhibition or dysfunction of HIPK2 in tumors impairs p53 function and activates oncogenic pathways necessary for tumor progression, angiogenesis, and resistance to chemotherapy [55, 56]. Thus, suppression of HIPK2 by shRNA or exosomal miR-1260b can induce angiogenesis and resistance to cisplatin, as shown in our data. However, resistance to cisplatin was not observed in Calu-1 cells. Several studies have suggested that the role of HIPK2 in sensitivity or resistance to chemotherapy is associated with p53-dependent apoptosis [57, 58]. Thus, the results in Calu-1 cells were likely caused by p53 because they have p53 deletion [59]. More experiments should be performed in various cells with p53 deletion.

MiR-1260b and HIPK2 showed an inverse relationship in NSCLC tissues, and high HIPK2 levels were associated with worse overall survival than low HIPK2 levels. In contrast, the levels of cellular miR-1260b were not associated with significant differences in survival rates. However, upregulation of exosomal miR-1260b was a poor prognostic marker, and high levels of exosomal miR-1260b were associated with worse overall survival than low levels, although the analysis of miR-1260b was performed in two independent cohorts. Furthermore, upregulation of exosomal miR-1260b was more evident in patients with late-stage NSCLC and metastasis. Thus, exosomal miR-1260b may be crucial in treating these tumors because patients with late-stage NSCLC have advanced/metastatic tumors and generally undergo chemotherapy. However, clinical significance of exosomal miR-1260b requires further exploration in a large cohort. In conclusion, exosomal miR-1260b induces angiogenesis, metastasis, and drug resistance by targeting HIPK2. Levels of cellular HIPK2 and exosomal miR-1260b may serve as prognostic biomarkers and may be applied as attractive therapeutic targets for NSCLC.

## Supplementary information

supplemetal data

## Data Availability

All data generated and analyzed during the current study are available from the corresponding author on reasonable request.
