# Supplementary material for: Exosomal miR-1260b derived from non-small cell lung cancer promotes tumor metastasis through the inhibition of HIPK2
Source: Cell Death Dis. 2021 Jul 28;12(8):747. doi: 10.1038/s41419-021-04024-9 (PMC8319168; doi:10.1038/s41419-021-04024-9)
Supplement: Supplementary file 1 — supplemetal data [file 41419_2021_4024_MOESM1_ESM.docx]

**Supplemental information**

**Exosomal miR-1260b derived from non-small cell lung cancer promotes tumor metastasis through the inhibition of HIPK2**

Dong Ha Kim^1^, Hyojeong Park^2^, Yun Jung Choi^1^, Myoung-Hee Kang^1^, Tae-Keun Kim^1^, Chan-Gi Pack^3^, Chang-Min Choi^4,5^, Jae Cheol Lee^5,a,*^ Jin Kyung Rho^3,a,*^

^1^Asan Institute for Life Sciences, Asan Medical Center, University of Ulsan, College of Medicine, Seoul 05505, South Korea.

^2^Department of Biomedical Sciences, Asan Medical Center, AMIST, University of Ulsan, College of Medicine, Seoul 05505, South Korea.

^3^Department of Convergence Medicine, Asan Medical Center, University of Ulsan, College of Medicine, Seoul 05505, South Korea.

^4^Department of Pulmonology and Critical Care Medicine, Asan Medical Center, University of Ulsan, College of Medicine, Seoul 05505, South Korea.

^5^Department of Oncology, Asan Medical Center, University of Ulsan, College of Medicine, Seoul 05505, South Korea.

^a^These authors contributed equally to this study.

*Co-corresponding authors:

Jae Cheol Lee

Department of Oncology, University of Ulsan College of Medicine, 88, Olympic-ro 43-gil, Songpa-gu, Seoul 05505, South Korea. Tel: +82 2 3010 3208; Fax: +82 2 3010 6961; E-mail: [jclee@amc.seoul.kr](mailto:jclee@amc.seoul.kr)

Jin Kyung Rho

Department of Convergence Medicine, University of Ulsan College of Medicine, 88, Olympic-ro 43-gil, Songpa-gu, Seoul 05505, South Korea. Tel: +82 2 3010 2974; Fax: +82 2 3010 6961; E-mail: [jkrho@amc.seoul.kr](mailto:jkrho@amc.seoul.kr)


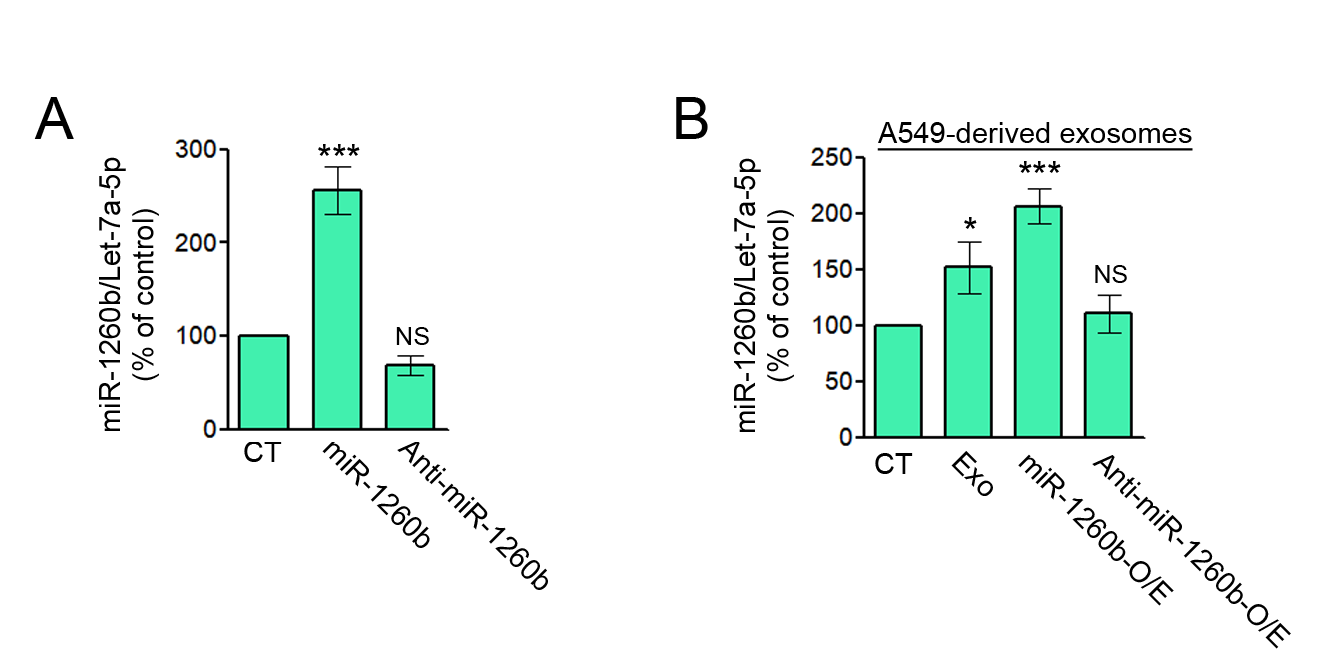


**Fig. S1. Expression levels of miR-1260b in HUVECs.** Changes in miR-1260b levels were determined using qRT-PCR in Fig. 1A and 1D. Let-7a-5p was used as an internal control. All data are reported as the mean ± standard deviation. **P* < 0.05, ****P* < 0.0005 compared with the control group.


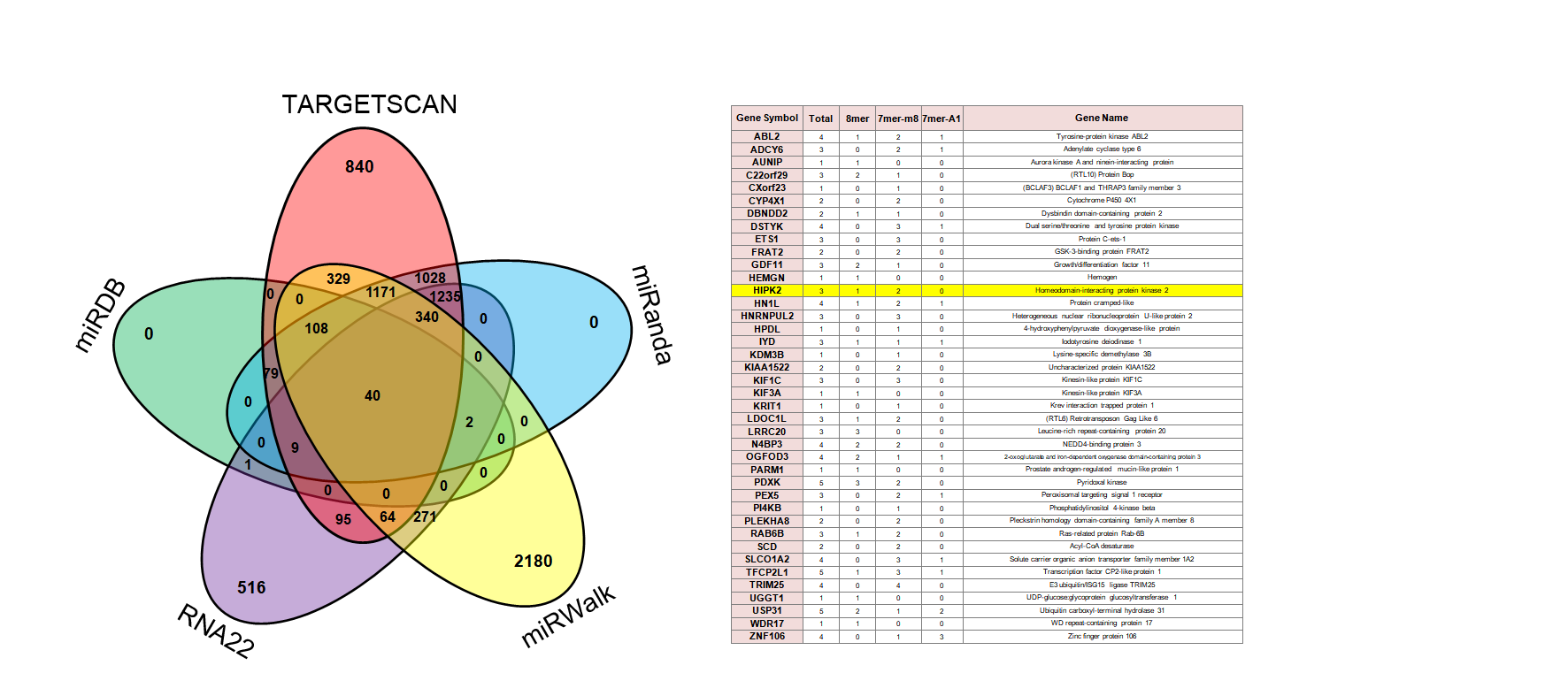


**Fig. S2. Prediction of targeted mRNA by miR-1260b.** Candidate gene targets were predicted by intersecting outputs from five distinct prediction algorithms (TargetScan, miRanda, miRWalk, RNA22, and miRDB). The potential miR-1260b seed region in the 40 predicted target genes was computationally predicted.


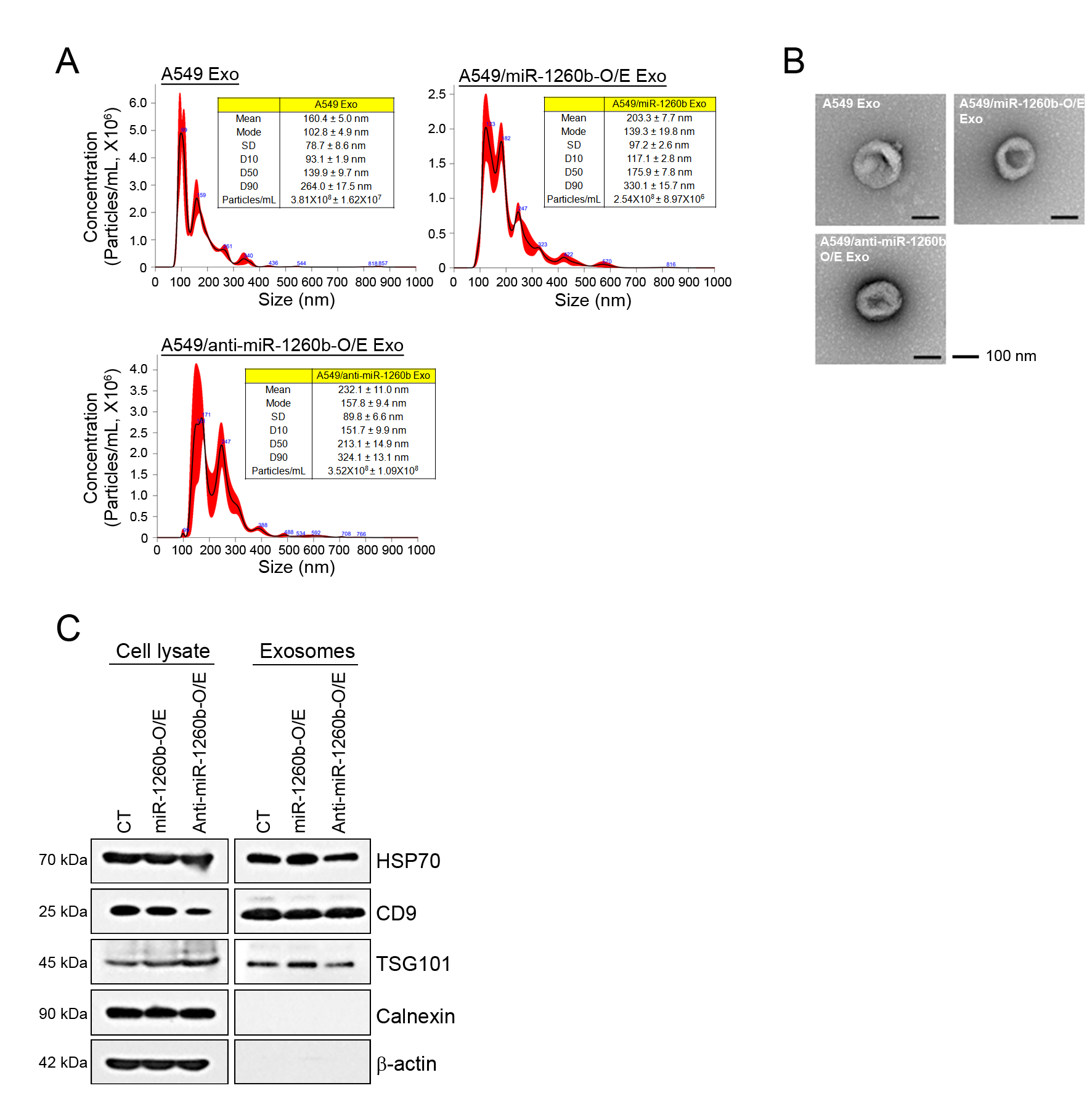


**Fig. S3. Characterization of exosomes.** Exosomes derived from each cell (A549 and miR-1206b-overexpressing or anti-miR-1260b-overexpressing A549 cells) were isolated using ultracentrifugation, as described in the Materials and Methods section. (A) Size distribution of the isolated particles determined using nanoparticle tracking analysis (NTA). (B) Examination of isolated exosomes by transmission electron microscopy. (C) Western blotting of exosomal proteins performed to validate the expression of exosome biomarkers.


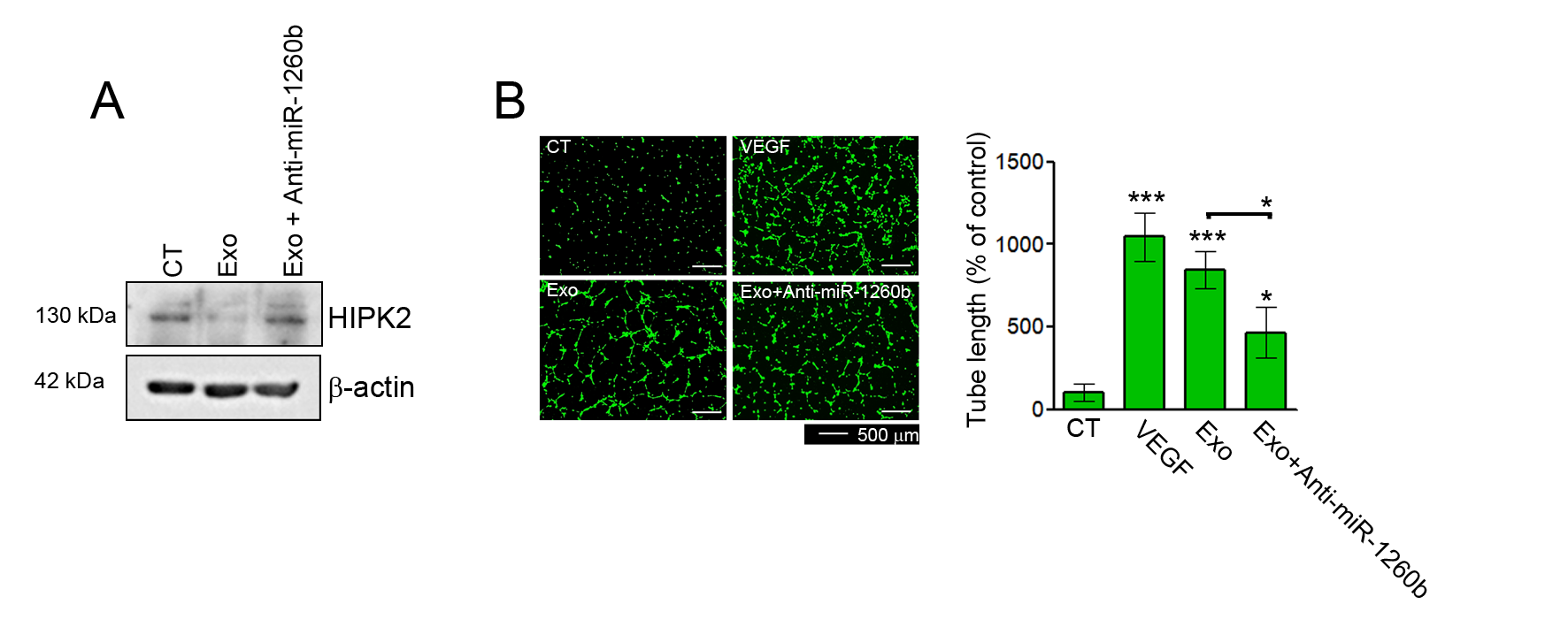


**Fig. S4. Effects of exosomal miR-1260b on angiogenesis.** HUVECs were treated with 50 μg of exosomes derived from A549 following the transfection of anti-miR-1260b. (A) HIPK2 expression was confirmed by western blotting. (B) The tube formation ability was determined by tube length. All data are reported as the mean ± standard deviation. **P* < 0.05, ****P* < 0.0005 compared with the control group.


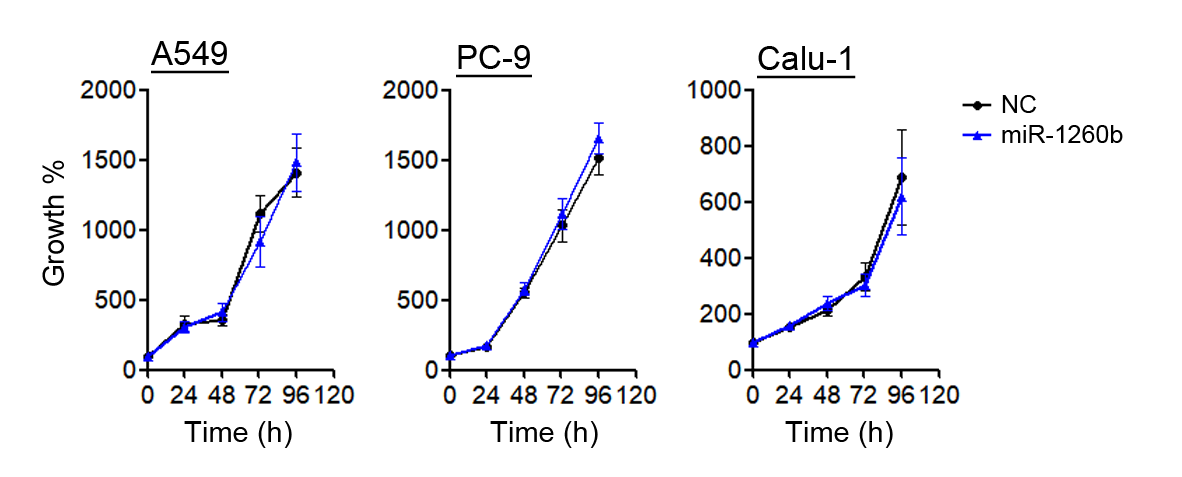


**Fig. S5. Proliferation effects of miR-1260b in NSCLC cells.** The cells were transfected with control miRNA (NC) or miR-1260b mimic, and cell proliferation was measured by using CCK-8 at the indicated time points.


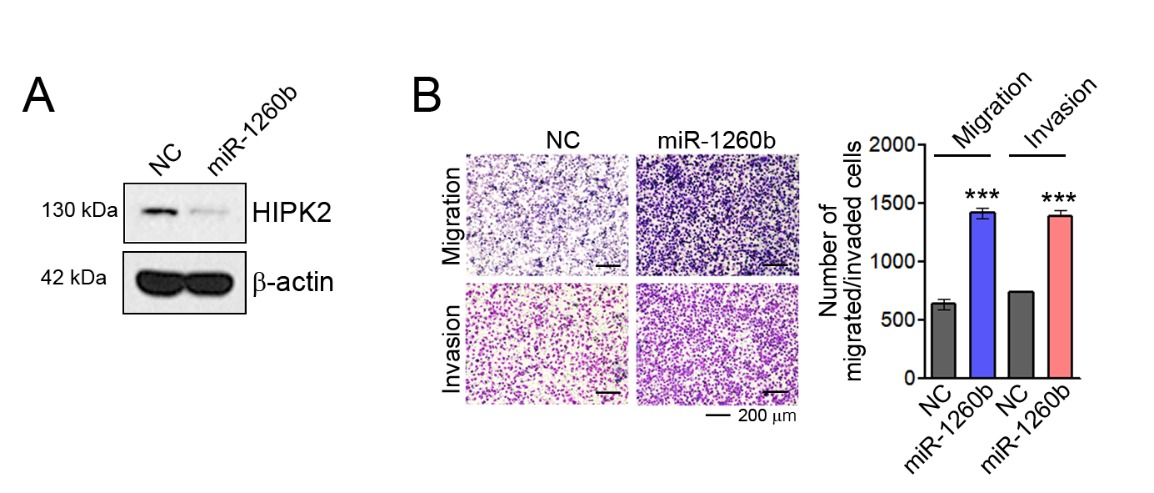


**Fig. S6.** **Effects of miR-1260b on migration and invasion of PC-9 cells.** PC-9 cells were transfected with 50 nM control miRNA (NC) or miR-1260b mimic for 48 h. (A) HIPK2 protein expression was confirmed by western blotting. (B) Transwell assays were performed to detect changes in migration and invasion abilities. The number of migratory or invading cells was counted for each image field. Data are reported as the mean ± standard deviation of three independent experiments with five fields counted per experiment. ****P* < 0.0005 compared with the control group.

| Table S1. Clinicopathologic characteristics of 124 patients | | | |  |  |  |  |
| --- | --- | --- | --- | --- | --- | --- | --- |
|  |  |  |  |  |  |  |  |
|  | Total | HIPK2 | |  | miR-1260b | |  |
| Variable | (n=124) | Low (n=62) | High (n=62) | *P*-value | Low (n=62) | High (n=62) | *P*-value |
| Age |  |  |  | 0.09 |  |  | 0.138 |
| ≤60 years | 45 | 44 (71.0%) | 27 (43.5%) |  | 22 (35.5%) | 23 (%) |  |
| >60 years | 79 | 18 (29.0%) | 35 (56.5%) |  | 40 (64.5%) | 39 (%) |  |
| Sex |  |  |  | 0.07 |  |  | 0.429 |
| Male | 100 | 54 (87.1%) | 46 (74.2%) |  | 47 (75.8%) | 53 (%) |  |
| Female | 24 | 8 (12.9%) | 16 (25.8%) |  | 15 (24.2%) | 9 (%) |  |
| Histology |  |  |  | 0.366 |  |  | 0.181 |
| SCC | 51 | 28 (45.2%) | 23 (37.1%) |  | 28 (45.2%) | 23 (37.1%) |  |
| AC | 73 | 34 (54.8%) | 39 (62.9%) |  | 34 (54.8%) | 39 (62.9%) |  |
| Tumor invasion |  |  |  | 1 |  |  | 0.182 |
| T1/T2 | 78 | 39 (62.9%) | 39 (62.9%) |  | 40 (64.5%) | 24 (38.7%) |  |
| T3/T4 | 46 | 23 (37.1%) | 23 (37.1%) |  | 22 (35.5%) | 38 (61.3%) |  |
| Lymph node metastasis | |  |  | 0.279 |  |  | **0.001** |
| Negative | 52 | 23 (37.1%) | 29 (46.8%) |  | 27 (43.5%) | 17 (27.4%) |  |
| Positive | 72 | 39 (62.9%) | 33 (53.2%) |  | 35 (56.5%) | 45 (72.6%) |  |
| Distant metastasis |  |  |  | **0.04** |  |  | **0.026** |
| Negative | 111 | 52 (83.9%) | 59 (95.2%) |  | 57 (91.9%) | 54 (87.1%) |  |
| Positive | 13 | 10 (16.1%) | 3 (4.8%) |  | 5 (8.1%) | 8 (12.9%) |  |
| Stage |  |  |  | 0.151 |  |  | 0.079 |
| Ⅰ+Ⅱ | 56 | 24 (38.7%) | 32 (51.6%) |  | 32 (51.6%) | 24 (38.7%) |  |
| Ⅲ+Ⅳ | 68 | 38 (61.3%) | 30 (48.4%) |  | 30 (48.4%) | 38 (61.3%) |  |
|  |  |  |  |  |  |  |  |

| Table S2. Primer sequences | |
| --- | --- |
|  |  |
| Primer name | Primer sequences |
| Primers for quantification of mRNA |  |
| HIPK2 (Forward) | 5’-GGCCTCACATGTGCAAGTTTTC-3’ |
| HIPK2 (Reverse) | 5’-TTGGTAGGTATCAAGGAGGCTC-3’ |
| Beta-actin (Forward) | 5’-TTGTTACAGGAAGTCCCTTGCC-3’ |
| Beta-actin (Reverse) | 5’-ATGCTATCACCTCCCCTGTGTG-3’ |
| Primers for quantification of miRNA |  |
| Poly(T) adaptor | 5’-GCGAGCACAGAATTAATACGACTCACTATAGG(T)12VN-3’ |
| Adaptor (Reverse) | 5’-GCGAGCACAGAATTAATACGAC-3’ |
| miR-1260b (Forward) | 5’-ATCCCACCACTGCCACCAT-3’ |
| let-7a-5p (Forward) | 5’-TGAGGTAGTAGGTTGTATAGTT-3’ |
